# How does digital trade promote and reallocate the export technology complexity of the manufacturing industry? Evidence from 30 Chinese provinces, 2011–2020

**DOI:** 10.1371/journal.pone.0291464

**Published:** 2023-09-21

**Authors:** Yaobin Liu, Weihui Hu, Kang Luo, Yan Guo, Zichang Wang

**Affiliations:** 1 School of Economics and Management, Nanchang University, Nanchang, Jiangxi, China; 2 School of Economics, Hainan University, Haikou, Hainan, China; East China Normal University, CHINA

## Abstract

It is important for China to break the “low-end lock” of the manufacturing value chain worldwide by revealing how digital trade promotes and reallocates the export technology complexity of the manufacturing industry. Panel data for 30 provinces in China from 2011 to 2020 were employed to measure the digital trade development and export technology complexity of the manufacturing industry. Benchmark regression, intermediary effect regression, panel threshold and other models were used to test the promotion and reallocation of digital trade on the export technology complexity of the manufacturing industry. The findings are as follows: (1) Digital trade promotes the export technology complexity of the manufacturing industry, with significant regional heterogeneity (eastern, central and western regions), and the most obvious promotion in technology-intensive manufacturing. (2) Technological innovation and human capital play a reallocation role in the process of digital trade, affecting the technological complexity of manufacturing exports, with mediating effects of 14.19% and 8.61%, respectively. (3) Digital trade promotes and reallocates the export technology complexity of the manufacturing industry through industrial structure upgrading, and a nonlinear relationship was found. These results provide empirical support and a decision-making basis for digital trade in promoting the export technology complexity of the manufacturing industry. The development of digital trade should be encouraged; the differential development of digital trade in the eastern, central, and western regions should be boosted; importance should be attached to the intermediary incentive role of technological innovation and human capital; and the upgrading of the industrial structure should be promoted scientifically.

## Introduction

With the advent and widespread adoption of information technology, global digital trade has experienced rapid growth, fundamentally reshaping the competitive landscape of global trade and readjusting the division of value chains. According to a report by the United Nations Conference on Trade and Development, the share of global digital services trade in overall services trade increased from 48.0% in 2011 to 63.6% in 2020. Historically, China’s foreign trade development has relied primarily on cost advantages rather than innovation and quality differentiators. However, recent policy initiatives have recognized digital trade as a new driving force for China’s foreign trade development. The State Council has issued guidelines promoting innovative foreign trade development, with an emphasis on fostering digital commerce. During the 20th National Congress, it was proposed that efforts toward establishing China as a strong trading nation be accelerated. China has a high Internet penetration rate, relatively rich data resources, and a wealth of trade digital application scenarios, which have laid a solid foundation for the development of digital trade. China’s digital trade volume rose from $200 billion in 2015 to $295 billion in 2020, an increase of 47.4%. Consequently, China has emerged as a significant player in international digital trade.

With the advancement of global digital trade, worldwide merchandise exports are projected to surge from US $6,196 billion in 2001 to US $17,645 billion by 2020, exhibiting an average annual growth rate of 6%. Concurrently, China’s export volume has witnessed a remarkable escalation, from 2.20 trillion yuan in 2001 to 17.93 trillion yuan in 2020, with an average annual growth rate of 11.7%. However, with international industrial transfer and the rise of trade protectionism, China’s export trade needs to change from “winning by quantity” to “winning by quality.”

Manufacturing serves as the cornerstone of a nation’s comprehensive prowess. As per China’s Ministry of Industry and Information Technology data, the national industrial added value reached 31.31 trillion yuan in 2020, securing the top position in global manufacturing for an uninterrupted span of 11 years. However, the “two ends outside” development model has yielded substantial economic gains for China while perpetuating its prolonged positioning within the middle and lower echelons of the international value chain. It is important to delve into how digital trade influences export trade, particularly with regard to manufacturing exports.

Digital trade facilitates factor flow, enhances the accumulation of factor capital such as technology and human resources, and optimizes production allocation [1]. It redefines factors of production like innovation and labor by significantly reducing trade costs and boosting export growth [2]. This raises the questions of whether digital trade increases the complexity of manufacturing export technologies and whether technological innovation and human capital contribute to reallocation. This is a pivotal topic.

Limited research has focused on provinces as the subject of study, meaning that the potential effects of heterogeneity remain neglected. There is a scarcity of literature exploring the potential mediating impact of digital trade on the technological complexity of manufacturing exports, making it challenging to fully elucidate the influencing mechanism. To address this gap, this paper aims to expand the existing research in two ways. First, it establishes an index evaluation system that measures digital trade development from the perspective of “infrastructure–industrial scale–development potential.” When measuring the technical complexity of manufacturing exports, differences in regional economic development were fully considered, which compensates for the shortcomings of the traditional evaluation system. Second, it uncovers the significant mediating roles played by technological innovation and human capital in shaping how digital trade influences the technological complexity of manufacturing exports, along with highlighting the nonlinear effects resulting from industrial structure upgrading.

The rest of this paper is organized as follows. Section 2 briefly reviews the existing research on digital trade and manufacturing export technology complexity, and advances the hypothesis of this paper. Section 3 describes the data and methodology of this study. Section 4 discusses the empirical results and further analyses. Section 5 provides the conclusions and implications.

## Literature review and hypothesis development

### Literature review

The definition of digital trade comes from the digital economy. It is believed that the development of the digital economy has promoted the innovation of various products in the industry and has had a profound impact on the global trade system, thus forming digital trade [3, 4]. According to Weber, digital trade refers to trade involving the transmission of valuable products or services through electronic delivery, with the core being digital products or services [5]. Deardorff defined digital trade as a business involving multiple countries in which the products traded are digital [6]. At present, most research on digital trade measurement incorporates the report of typical digital enterprises, the descriptive statistics of relevant institutions, and the comparison of transnational data [7, 8]. Some scholars have also established a digital trade evaluation index system including Internet development, trade potential, and legal supervision to evaluate major countries [9].

The assessment of export technical complexity relies primarily on the approach developed by Hausmann et al. [10] and its subsequent evolution and expansion, which has been widely adopted in research. For instance, Fan et al. [11] contended that cultural diversity enhances the intricacy of exports, while Thorbecke and Salike [12] compared the export technological complexity among major exporting nations from the perspective of trade resilience. Su et al. [13], using macro panel data spanning 2005–2014 for 36 countries, demonstrated that service trade restrictions exerted an adverse effect on export complexity.

Worldwide, the level of digital trade development varies greatly between developed and developing economies [14, 15]. Due to their relatively advanced and mature technology development, European and American countries have always occupied the first echelon of global trade. However, in some regions, such as Latin America, digital trade remains on the fringes of the Internet and trading system [16], which significantly inhibits import—export processes. Nevertheless, scholars propose that promoting digital technology and trade integration can reduce input costs for enterprises while enhancing their export advantages [17]. Digital trade reduces search costs, information costs, and overall trading expenses while promoting export growth [18]. Based on its unique characteristics as a digital element itself, it also improves innovation levels in product production/sales, thus improving the competitiveness of exported products. We view this process as a promotion.

Some studies have stated that in the process of digital transformation, increasing R&D investment can help improve the forward-looking participation of a country’s manufacturing industry in the global value chain [19]. The integration of innovation research and development and manufacturing in the process of digital trade can promote the research and development process of market demand, improve the domestic added value of product exports, and ensure the quality improvement of product exports [20].

At the same time, some scholars have found that the complementarity between digital technology and human resources in production blocks facilitates the flow of bilateral online trade [21]. For people with higher international integration, the redistribution effect of labor allocation is more obvious, especially the gap in labor productivity within the manufacturing industry. For example, ICT affects the redistribution of workers between skilled and unskilled positions, which changes the technical complexity of a country’s manufacturing exports [22, 23].

In the development process of digital trade, with the emergence of new features of digital trade, various production factors, such as technology, labor, and capital, are added to production, sales, and other links, promoting the accumulation of R&D investment in technological innovation, and carrying out labor dislocation matching in human capital, which ultimately affects the export and complexity of manufacturing trade. We view this process as reallocation.

In summary, scholars have made some progress in research on digital trade and export technology complexity, but there is no complete and accurate public document on the measurement of digital trade. The measurement manual of digital trade is still at the initial stage in the application of measurement, and there are difficulties in accounting. At present, research on export technology complexity focuses less on the relevant measurement of provinces and manufacturing sectors. Against the background of the innovation of digital technology and the rapid development of global digital trade, few studies have explored the internal correlation mechanism and impact path of digital trade on the export technology complexity of the manufacturing industry. As a new breakthrough for exports in the digital era, can digital trade achieve a leap and break the “low-end lock” in China’s manufacturing value chain? This question is the focus of this paper.

### Hypotheses

Digital trade has brought potential opportunities to the global economy, trade, and enterprises. For example, Xing [24] empirically found that the application of modern ICT and e-commerce stimulated bilateral trade flows based on data from 21 developing and least developed countries and 30 OECD countries. Goldfarb and Tucker [25] found that digital trade promotes the integration of enterprise information resources, drives the transformation of enterprise production and R&D processes, and improves enterprise management efficiency. Compared with traditional enterprises, enterprises with digital backgrounds have higher technological levels in the process of R&D, production, and trade, and higher export technology complexity [26].

With the deepening of specialization, digital trade effectively reduces production and transaction costs, promotes the improvement of manufacturing production level and efficiency, and thus increases the export technology complexity of the regional manufacturing industry [27]. The transformation of digital technology has changed the traditional trade structure, realized the efficient conversion of traditional physical goods and digital products through the transformation of industrial Internet, and further improved the export technology complexity of the manufacturing industry [28]. Digital trade effectively breaks spatio-temporal constraints, reduces trade search and transaction costs, and encourages more enterprises to participate in national trade for learning and division of labor, which has a direct impact on improving the export technology complexity of the manufacturing industry [29]. Therefore, the first hypothesis in this paper is as follows:

Hypothesis 1. *Digital trade directly and significantly promotes the export technology complexity of the manufacturing industry*, *thus having a promoting effect*.

Compared with traditional trade, the characteristics of digital trade enable it to be conducted through digital transmission, fundamentally changing the inefficiency and non-tradability of traditional service industries, effectively reducing intermediate input costs, and improving trade efficiency [30]. Compared with capital allocation efficiency, the technology application of digital trade plays a more obvious role in promoting factor allocation efficiency. An increase in innovation input and output is conducive to an increase in enterprises’ total exports [31], and product innovation has a significant impact on the increase in enterprises’ export tendencies [32]. At the same time, in the context of digital trade, trade redistributes the factors of production between low- and high-tech economic [33]. Due to the existence of market entry barriers, manufacturing exports have a “self-selection” effect, and only highly skilled subjects can choose manufacturing exports. As the input of production factors, technological innovation has gradually replaced low-end production factors, such as low-skilled labor, while changing the traditional trade mode. In this process, digital trade has a positive technology spillover effect and promotes an increase in the technological complexity of enterprises [34]. Therefore, Hypothesis 2a is as follows:

Hypothesis 2a. *The technological innovation effect is the main path for digital trade to promote the export technology complexity of the manufacturing industry*, *and it plays the role of reallocation in this process*.

The development of digital trade can accelerate the transmission and sharing of information and knowledge, promote the learning and production efficiency of workers at all levels, continuously improve the education level of enterprise employees, and reshape the efficiency of labor adjustment [35–37].

Human capital has a stronger positive impact on the product innovation of enterprises located in clusters with more derivatives [38]. At the same time, regions with higher levels of human capital tend to have higher productivity, and are more likely to have comparative advantages [39]. They can promote the export of multiple types of inputs by processing trade enterprises, stimulate investment in fixed assets, and improve product technology complexity. Therefore, Hypothesis 2b is as follows:

Hypothesis 2b. *The human capital effect is the main path for digital trade to promote the export technology complexity of the manufacturing industry*, *and it plays a role of reallocation in this process*.

Digital trade is a new driving force for reshaping the traditional value chain and promoting industrial transformation and upgrading [40]. The impact of the digital transformation of the manufacturing industry on its export technology complexity has significant nonlinear characteristics. Digital trade itself is applicable to the network marginal effect [41]. With an increasing number of participants, the value of digital trade shows nonlinear growth, which is reflected in a marginal decline in production costs and an increase in scale economies. The change in industrial structure in the development of digital trade is dynamic and continuous [42]. At the beginning of digital trade development, relatively higher construction and operation costs make it difficult to support the capital needed for the change of the regional industrial structure. At this point, the export of the manufacturing industry is still dominated by industries with comparative labor advantages. When more advanced digital and information factors enter the manufacturing production chain, the injection of production factors directly changes the regional industrial structure. Moreover, because the region needs to obtain comparative advantages, new requirements are indirectly put forward for the change of the regional industrial structure, jointly promoting the export technology complexity of the manufacturing industry. Therefore, Hypothesis 3 is as follows:

Hypothesis 3. *Digital trade promotes and reallocates the export technology complexity of the manufacturing industry by industrial structure upgrading*, *but it is a threshold process*.

**Fig 1:**
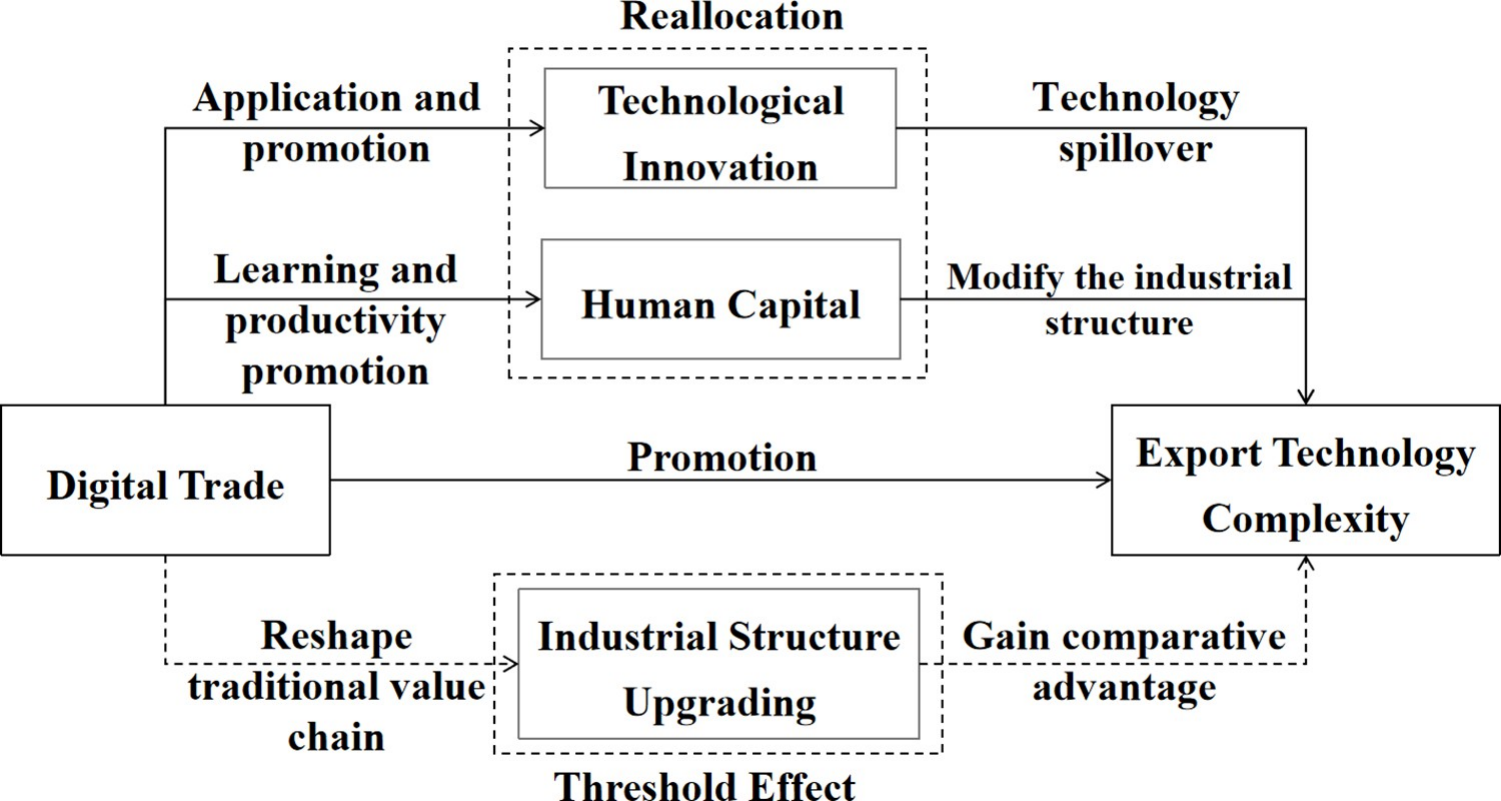
Theoretical mechanism of digital trade affecting the export technology complexity of the manufacturing industry.

Based on the panel data of 30 Chinese provinces from 2011 to 2020, a theoretical mechanism diagram is plotted in terms of the impact of digital trade on the export technology complexity of the manufacturing industry (Fig 1).

## Data and methodology

### Variables

#### Dependent variable

*Export technology complexity of manufacturing industry (etc)*. The commonly used export technology complexity index is derived from the calculation method developed by Hausmann et al. [10], based on comparative advantage theory, and the positive relationship between export technology complexity and regional economic development is taken into account. This paper focuses on the export technology complexity of the manufacturing industry. Based on relevant literature [43], in this paper, special trading and miscellaneous products that do not accurately reflect the change in export technology structure were removed from the 22 export products under the HS code of China Customs, resulting in 12 manufacturing export products (Table 1).

**Table 1:** Manufacturing export products and codes.

Customs code	Commodity code	Commodity name
T06	Category VI	Products of chemical industry and related industries
T07	Category VII	Plastics and their products; Rubber and its products
T08	Category VIII	Hides, leather, furs and their products; Saddle and harness; Travel supplies, handbags and similar containers; Animal catgut (except silkworm gut) products
T09	Category IX	Wood and woodware; Charcoal; Cork and cork products; Straw, stipa capillata or other woven products; Basket and wicker plaited cork products
T10	Category X	Wood pulp and other fibrous cellulose pulp; Recycled (scrap) paper or paperboard; Paper, paperboard and their products
T11	Category XI	Textile raw materials and textile products
T12	Category XII	Shoes, hats, umbrellas, sticks, whips and their parts; Processed feathers and their products; Artificial flowers; Human hair products
T13	Category XIII	Stone, gypsum, cement, asbestos, mica and similar materials; Ceramic products; Glass and its products
T15	Category XV	Base metals and their products
T16	Category XVI	Machines, mechanical appliances, electrical equipment and their parts; Recording and reproducing equipment and its parts and accessories for tape recorders and sound players, television images and sound
T17	Category XVII	Vehicles, aircraft, ships and related transportation equipment
T18	Category XVIII	Optical, photographic, cinematographic, metrological, inspection, medical or surgical instruments and equipment, precision instruments and equipment; Clocks and watches; Musical instruments; Parts and accessories of the above items

First, the export technology complexity of each type of export product is calculated, and the formula is as follows:

$$petc_{kt}=\sum_{i}\frac{\frac{x_{ikt}}{X_{it}}}{\sum_{i}\frac{x_{ikt}}{X_{it}}}Y_{it}$$ (1)

where petc_kt_ is the export technology complexity of category k products in year t, x_ikt_ is the export volume of category k products in i province (city) in year t, X_it_ is the total export volume in i province (city) in year t, and Y_it_ is the actual per capita GDP of i province (city) in year t (taking 2011 as the base period and excluding price factors).

Second, the weighted average of the export technology complexity of the corresponding categories of products is calculated based on the proportion of exports of various products as the weight to obtain the export technology complexity of the manufacturing industry in each province and city:

$$etc_{it}=\sum_{k}\frac{x_{ikt}}{X_{it}}petc_{kt}$$ (2)

where etc_it_ is the export technology complexity of the manufacturing industry in i province (city) in year t.

#### Independent variable

*Digital trade development level (dt)*. According to the concept of digital trade [9], starting from “infrastructure—industrial scale—development potential,” an evaluation index system of digital trade development level composed of 3 first-level indicators and 11 second-level indicators is constructed (Table 2). In this paper, the entropy method is used to objectively analyze the original information and determine the index weight, which avoids the limitation of subjective weight to some extent. The specific calculation steps are as follows:

**Table 2:** Evaluation index system of digital trade development level.

First-level index	Second-level index	Unit and attribute	Weight
Digital trade infrastructure (0.3799)	Number of domain names	10,000 (+)	0.1208
	Number of websites	10,000 (+)	0.1111
	Internet broadband access ports	10,000 (+)	0.0526
	Number of enterprises with e-commerce trading activities	1 (+)	0.0955
Scale of digital trade-related industries (0.5287)	Telecommunication services	100 million yuan (+)	0.1104
	Software business income	10,000 yuan (+)	0.1550
	Software product income	10,000 yuan (+)	0.1474
	E-commerce sales	100 million yuan (+)	0.1159
Development potential of digital trade (0.0914)	GDP per capita	1 yuan (+)	0.0334
	Per capita disposable income	1 yuan (+)	0.0378
	Number of college students per 100,000 persons	1 person (+)	0.0202

Note: The data are originated from *China Statistical Yearbook* from 2012 to 2021.

To eliminate the impact of the index dimension on the evaluation results, the range method is used to standardize the data of each evaluation index, and the standardization matrix is constructed. The forward and contrary indexes are processed as follows:

$$r_{ij}=\frac{x_{ij}-\min(x_{ij})}{\max(x_{ij})-\min(x_{ij})}$$ (3)

$$r_{ij}=\frac{\max(x_{ij})-x_{ij}}{\max(x_{ij})-\min(x_{ij})}$$ (4)

Determine the index weight: normalize and calculate the information entropy of the ith index to obtain the weight w_i_ of each index:

$$p_{ij}=\frac{r_{ij}}{\sum_{j=1}^{n}r_{ij}}$$ (5)

$$e_{i}=-k\sum_{j=1}^{n}p_{ij}\;\ln\;p_{ij},k=\frac{1}{\ln\;n}>0,0\cdot \ln\;0\equiv 0$$ (6)

$$g_{i}=1-e_{i},w_{i}=\frac{g_{i}}{\sum_{i=1}^{m}g_{i}}$$ (7)

Obtain the comprehensive evaluation index of the digital trade development level:

$$dt=\sum_{i=1}^{n}r_{ij}w_{i}$$ (8)

### Mediating variables and other variables

#### (1) Mediating variables

According to Hypotheses 2a and 2b, technological innovation and human capital are selected as intermediary variables.

Technological innovation (pat): There is a long-term co-integration relationship between technological innovation and the export technology complexity of the manufacturing industry [44, 45]. Compared with the number of patent applications, the number of patent authorizations can more accurately reflect regional innovation capacity. Therefore, the logarithmic number of patent authorizations is adopted to measure technological innovation.

Human capital (hum): The population is divided into illiterate and semi-illiterate, primary school, junior high school, senior high school, technical secondary school, junior college, and above, according to education level, and the level of human capital is calculated by weighted average. According to Hypothesis 3, industrial structure upgrading is selected as the threshold variable.

Industrial structure upgrading (ind): This is expressed by the ratio of the added value of the tertiary industry to that of the secondary industry.

#### (2) Control variables

This paper refers to the relevant literature and selects the control variables as follows.

Industrial capital intensity (ci): The higher the industrial capital intensity, the more intense the competition in the industry, and the stronger the willingness to pursue cost reduction. This helps expand exports [46]. The ratio of fixed asset investment in the manufacturing industry to the number of manufacturing employees is used to measure industrial capital intensity.

Labor productivity (lp): The improvement of labor productivity contributes to the reduction of domestic trade costs [47]. The ratio of manufacturing added value to the number of manufacturing employees is used to measure labor productivity.

Foreign trade openness (open): Trade liberalization can positively promote the export technology complexity of a country or region, causing FDI to have a positive incentive effect on export technology complexity [48]. The proportion of total foreign direct investment to GDP is used to measure this index.

Domestic market openness (mar): This is sourced from the Marketization Index of China’s Provinces (2006–2018), and the data of subsequent missing years are supplemented by the historical average method.

Industrial wage level (wl): The ratio of manufacturing employees’ income to the number of manufacturing employees is used to measure this index.

### Empirical models

To clarify the impact mechanism of digital trade on the export technology complexity of the manufacturing industry, the following econometric model was established based on the above theoretical analysis:

$$etc_{it}=\alpha_{0}+\alpha_{1}\cdot dt_{it}+\sum k_{i}cv_{ijt}+\mu_{i}+\gamma_{t}+\varepsilon_{it}$$ (9)

Formula (9) expresses the benchmark regression model of digital trade development to the export technology complexity of the manufacturing industry, where i refers to each province and city, t refers to the year, and j refers to the number of control variables. etc_it_ is the explained variable of the export technology complexity of the manufacturing industry, dt_it_ is the core explanatory variable of digital trade development, α_0_ is a constant item, α_1_ and k_j_ are the coefficients to be estimated for the core explanatory variable dt_it_ and the control variable cv_ijt_ respectively, μ_i_ stands for individual fixed effect, γ_t_ is the time fixed effect, and ε_it_ is a random perturbation term.

After the model of the direct impact of digital trade on the export technology complexity of the manufacturing industry is established by Formula (9), then the impact of digital trade on technological innovation is examined with technological innovation as the explained variable:

$$pat_{it}=\beta_{0}+\beta_{1}\cdot dt_{it}+\sum k_{i}cv_{ijk}+\mu_{i}+\gamma_{t}+\varepsilon_{it}$$ (10)

Finally, the digital trade development level and the effect of technological innovation are included in the model at the same time to explore whether technological innovation has an intermediary effect between digital trade and the export technology complexity of the manufacturing industry:

$$etc_{it}=\gamma_{0}+\gamma_{1}\cdot dt_{it}+\gamma_{2}\cdot pat_{it}+\sum k_{i}cv_{ijt}+\mu_{i}+\gamma_{t}+\varepsilon_{it}$$ (11)

In the above formula, i and t represent provinces/cities and years, respectively, etc_it_ represents the export technology complexity of the manufacturing industry in i province (city) in year t, dt_it_ represents the digital trade development level in i province (city) in year t, pat_it_ represents the technological innovation effect in i province (city) in year t, and cv_ijt_ is a series of control variables, including industrial capital intensity (ci), industrial labor productivity (lp), foreign trade openness (open), and domestic market openness (mar). μ_i_ stands for individual fixed effect, γ_t_ is the time fixed effect, and ε_it_ is a random perturbation term.

After the model of the direct impact of digital trade on the export technology complexity of the manufacturing industry is established by Formula (9), then the impact of digital trade on human capital is examined with human capital as the explained variable:

$$hum_{it}=\beta_{0}+\beta_{1}\cdot dt_{it}+\sum k_{i}cv_{ijk}+\mu_{i}+\gamma_{t}+\varepsilon_{it}$$ (12)

Finally, the digital trade development level and human capital are included in the model at the same time to explore whether human capital has an intermediary effect between digital trade and the export technology complexity of the manufacturing industry:

$$etc_{it}=\gamma_{0}+\gamma_{1}\cdot dt_{it}+\gamma_{2}\cdot hum_{it}+\sum k_{i}cv_{ijt}+\mu_{i}+\gamma_{t}+\varepsilon_{it}$$ (13)

In the above formula, i and t represent provinces/cities and years, respectively, etc_it_ represents the export technology complexity of the manufacturing industry in i province (city) in year t, dt_it_ represents the digital trade development level in i province (city) in year t, hum_it_ represents the human capital effect in i province (city) in year t, and cv_ijt_ is a series of control variables, including industrial capital intensity (ci), industrial labor productivity (lp), foreign trade openness (open), and domestic market openness (mar). μ_i_ stands for individual fixed effect, γ_t_ is the time fixed effect, and ε_it_ is a random perturbation term.

The panel threshold model [49] is employed to explore whether there is a threshold effect in industrial structure upgrading. The model is set as follows:

$$etc_{it}=\theta_{0}+\theta_{1}\cdot dt_{it}I(ind\leq \gamma )+\theta_{2}\cdot dt_{it}I(ind>\gamma )+\sum k_{i}cv_{ijt}+\varepsilon_{it}$$ (14)

$$\begin{array}{l} etc_{it}=\theta_{0}+\theta_{1}\cdot dt_{it}I(ind\leq \gamma_{1})+\theta_{2}\cdot dt_{it}I(\gamma_{1}<ind\leq \gamma_{2})\\ \;+\theta_{3}\cdot dt_{it}I(ind>\gamma_{2})+\sum k_{i}cv_{ijt}+\varepsilon_{it} \end{array}$$ (15)

$$\begin{array}{l} etc_{it}=\theta_{0}+\theta_{1}\cdot dt_{it}I(ind\leq \gamma_{1})+\theta_{2}\cdot dt_{it}I(\gamma_{1}<ind\leq \gamma_{2})\\ \;+\theta_{3}\cdot dt_{it}I(\gamma_{2}<ind\leq \gamma_{3})+\theta_{4}\cdot dt_{it}I(ind>\gamma_{3})+\sum k_{i}cv_{ijt}+\varepsilon_{it} \end{array}$$ (16)

As shown above, Formula (14) is a single threshold model, Formula (15) is a double threshold model, and Formula (16) is a triple threshold model. Formulas (14)–(16) are tested in turn until the threshold value of a model is not significant. i and t represent provinces and years respectively, etc_it_ represents the export technology complexity of the manufacturing industry in i province in year t, dt_it_ represents the digital trade development level in i province (city) in year t, ind_it_ represents the threshold variable of industrial structure upgrading, and cv_ijt_ is a series of control variables. θ is the regression coefficient, and I(·) is the indicative function. The value is 1 when the condition in brackets is true; otherwise, the value is 0. ε_it_ is a random perturbation term.

### Data description

Based on the availability and effectiveness of the data, the panel data of 30 provinces and cities in China from 2011 to 2020 are employed for empirical analysis, excluding those of Tibet, Hong Kong, Macao, and Taiwan. The relevant data on the export technology complexity of the manufacturing industry originate from the international trade research and decision support system of DRCnet (http://trade.drcnet.com.cn/data/goods/china/monthly). The data for core explanatory variables, intermediary variables, and control variables are from the China Statistical Yearbook (2012–2021), the China Statistical Yearbook of Science and Technology (2012–2021), and the statistical yearbooks of provinces (2012–2021). Individual missing data are supplemented by the interpolation method, the data involving prices are deflated in 2011 as the base period, and those involving monetary factors are converted by the historical exchange rate. To avoid the magnitude difference of each variable and the possible heteroscedasticity problem, some variables are logarithmized.

The descriptive statistical results of each variable are shown in Table 3. It can be seen that the size difference of some variables is relatively significant, indicating the unbalanced development of various provinces and cities in China. As seen in Table 4, there is a significant correlation between independent variable and dependent variable, and the correlation coefficient between variables does not exceed 0.8, so there is no obvious collinearity problem.

**Table 3:** Descriptive statistics of variables.

Variable	Sample size	Mean	Standard deviation	Minimum	Maximum
ln*etc*	300	9.959	0.574	8.169	11.100
*dt*	300	0.137	0.147	0.004	0.823
ln*pat*	300	10.110	1.439	6.219	13.470
*hum*	300	9.210	0.903	7.474	12.920
ln*ci*	300	12.830	0.784	10.820	14.130
ln*lp*	300	13.390	0.421	12.150	14.640
*open*	300	0.506	1.989	0.047	34.220
*mar*	300	6.824	2.024	2.330	11.880
ln*wl*	300	10.930	0.510	9.637	12.250
*ind*	300	1.325	0.730	0.527	5.297

**Table 4:** Correlation analysis of variables.

	ln*etc*	*dt*	ln*pat*	*hum*	ln*ci*	ln*lp*	*open*	*mar*	ln*wl*	*ind*
ln*etc*	1									
*dt*	0.273***	1								
ln*pat*	0.339***	0.774***	1							
*hum*	0.044	0.513***	0.378***	1						
ln*ci*	0.101*	-0.482***	-0.328***	-0.368***	1					
ln*lp*	-0.185***	-0.266***	-0.306***	0.101*	0.493***	1				
*open*	0.022	0.089	0.040	0.168***	-0.168***	-0.042	1			
*mar*	0.288***	0.766***	0.853***	0.592***	-0.360***	-0.292***	0.095*	1		
ln*wl*	-0.158***	0.256***	0.076	0.574***	0.059	0.596***	0.079	0.275***	1	
*ind*	-0.075	0.423***	0.101*	0.695***	-0.439***	0.07	0.246***	0.262***	0.512***	1

Note: ***,**, and * indicate statistically significant at 1%, 5%, and 10%, respectively.

## Empirical results

### Benchmark regression results

To avoid the influence of multicollinearity, the stepwise regression method is adopted to test the model. The regression results are shown in Table 5.

**Table 5:** Benchmark regression results.

	(1)	(2)	(3)	(4)	(5)
	ln*etc*	ln*etc*	ln*etc*	ln*etc*	ln*etc*
*dt*	2.769***	2.874***	2.827***	2.734***	1.410***
	(0.243)	(0.251)	(0.250)	(0.244)	(0.320)
ln*ci*		-0.100	-0.175**	-0.166**	-0.248***
		(0.062)	(0.071)	(0.069)	(0.067)
ln*lp*			0.254**	0.260**	0.313***
			(0.124)	(0.120)	(0.113)
*open*				0.0391***	0.0370***
				(0.009)	(0.009)
*mar*					0.199***
					(0.034)
*_cons*	9.579***	10.85***	8.415***	8.201***	7.383***
	(0.038)	(0.784)	(1.419)	(1.377)	(1.304)
Hausman	13.18***	18.38***	29.77***	34.89***	43.00***
Fixed effect	YES	YES	YES	YES	YES
*N*	300	300	300	300	300
adj. *R*^2^	0.251	0.255	0.264	0.308	0.387

Note: The standard error in parenthesis. ***,**, and * indicate statistically significant at 1%, 5%, and 10%, respectively.

As shown in Table 5, the overall fit of the model also increases with the increase in control variables, indicating that the explanatory power of the model is improving. As shown in column (1), digital trade has a significant positive impact on the export technology complexity of the manufacturing industry when no control variable is added. After the control variables are added gradually, its impact is still significantly positive, thus verifying Hypothesis 1.

In terms of control variables, the regression coefficient of industrial capital intensity is significantly negative, indicating that the current capital investment in the manufacturing industry has not effectively improved its export technology complexity. The regression coefficient of labor productivity is significantly positive, indicating that the improvement in labor productivity in the current manufacturing industry contributes to the improvement of its export technology complexity. The regression coefficient of the openness of foreign trade is significantly positive, indicating that a large amount of foreign investment can effectively promote the export technology complexity of the manufacturing industry. The regression coefficient of the openness of the domestic market is significantly positive, indicating that it openness of the domestic market contributes to the improvement of the export technology complexity of the manufacturing industry.

#### Robustness test of the benchmark model (Table 6)

We re-tested the robustness of the baseline regression in three ways. First, considering that there may be a two-way causal relationship between digital trade and the technical complexity of manufacturing exports, we took the first-order lag of the variable dt as an instrumental variable and used the two-stage least square method to estimate it. Second, using the entropy weight TOPSIS method to recalculate the development of digital trade, it can be seen that after changing the calculation method of explanatory variables, digital trade promotes the technical complexity of manufacturing exports, which is consistent with the above conclusion. Third, with regard to the outbreak of the new coronavirus pneumonia epidemic in 2020, although there are reports that the epidemic made online trade activities more active, many physical orders were unable to be performed normally, and the export scale of various provinces was affected. Therefore, the data for 2020 are deleted in this paper and regression estimates are performed on the benchmark model. The robustness test results are shown in Table 6. It can be seen that digital trade has a significant positive effect on the technical complexity of manufacturing exports.

**Table 6:** Results of robustness test of the benchmark model.

	Considering endogeneity	Replacing explanatory variable	Eliminating outliers
	ln*etc*	ln*etc*	ln*etc*
*dt*	1.297***	1.198***	1.363***
	(0.371)	(0.287)	(0.394)
ln*ci*	-0.265***	-0.268***	-0.185**
	(0.071)	(0.067)	(0.078)
ln*lp*	0.328***	0.343***	0.309**
	(0.120)	(0.113)	(0.129)
*open*	0.037***	0.037***	0.234
	(0.009)	(0.009)	(0.154)
*mar*	0.231***	0.213***	0.167***
	(0.041)	(0.032)	(0.037)
*_cons*	7.189***	7.184***	6.764***
	(1.403)	(1.302)	(1.392)
Fixed effect	YES	YES	YES
*N*	270	300	270
adj. *R*^2^	0.377	0.382	0.321

Note: The standard error in parenthesis. ***,**, and * indicate statistically significant at 1%, 5%, and 10%, respectively.

### Sensitivity analysis

Due to the vastness of territory in China, the development levels of provinces and cities in different regions are quite different. Therefore, 30 provinces and cities in China are classified into eastern, central, and western regions (The eastern region includes Beijing, Tianjin, Hebei Province, Shanghai, Jiangsu Province, Zhejiang Province, Fujian Province, Shandong Province, Guangdong Province, Hainan Province and Liaoning Province. The central region includes Shanxi Province, Anhui Province, Jiangxi Province, Henan Province, Hubei Province, Hunan Province, Jilin Province and Heilongjiang Province. The western region includes Inner Mongolia Autonomous Region, Guangxi Zhuang Autonomous Region, Chongqing, Sichuan Province, Guizhou Province, Yunnan Province, Shaanxi Province, Gansu Province, Qinghai Province, Ningxia Hui Autonomous Region and Xinjiang Uygur Autonomous Region) according to the administrative division standard of the National Bureau of Statistics for regression analysis, and the results are shown in Table 7.

**Table 7:** Results of the sensitivity analyses by region.

	(1)	(2)	(1)
	Eastern region	Central region	Western region
*dt*	1.690***	-0.448	-1.460
	(0.188)	(1.061)	(1.354)
ln*ci*	-0.271***	0.587***	-0.217
	(0.044)	(0.137)	(0.169)
ln*lp*	0.347***	-0.098	-0.078
	(0.096)	(0.202)	(0.227)
*open*	0.037***	1.028***	-0.818**
	(0.004)	(0.270)	(0.352)
*mar*	0.118***	0.112	-0.053
	(0.025)	(0.080)	(0.077)
*_cons*	7.323***	2.675	13.781***
	(1.291)	(2.146)	(2.102)
*N*	110	80	110
adj. *R*^2^	0.824	0.579	0.551

Note: The standard error in parenthesis. ***,**, and * indicate statistically significant at 1%, 5%, and 10%, respectively.

As shown in columns (1)–(3), only digital trade in the eastern region has a significant positive impact on the export technology complexity of the manufacturing industry, while digital trade in the central and western regions has no significant impact on export technology complexity, and the regression coefficient is negative. There is still a gap between the digital development level in the central and western regions and that in the eastern regions. Affected by natural terrain, low efficiency of resource allocation, backward digital infrastructure, and other factors, digital trade has not significantly promoted the export technology complexity of the manufacturing industry, but has had a restraining effect to some extent, because digital trade in the central and western regions has undergone phased and lagged development. It needs to reach a certain level before it can play its full role.

The technological levels and characteristics of the manufacturing industry are different. From the perspective of manufacturing industry classification, the sample is further divided into labor-intensive, capital-intensive, and technology-intensive (Capital-intensive: T06, T07, T13, T15; Labor-intensive: T08, T09, T10, T11, T12; Technology-intensive: T16, T17, T18). The regression results are shown in Table 8.

**Table 8:** Results of the sensitivity analyses by industry.

	(1)	(2)	(1)
	Labor-intensive	Capital-intensive	Technology-intensive
*dt*	0.792*	1.523***	1.602***
	(0.419)	(0.353)	(0.444)
ln*ci*	-0.109	-0.256***	-0.295***
	(0.087)	(0.073)	(0.092)
ln*lp*	0.269*	0.320**	0.324**
	(0.148)	(0.125)	(0.157)
*open*	0.049***	0.032***	0.039***
	(0.011)	(0.010)	(0.012)
*mar*	0.128***	0.149***	0.295***
	(0.044)	(0.037)	(0.047)
*_cons*	5.080***	6.762***	6.038***
	(1.705)	(1.435)	(1.808)
*N*	300	300	300
adj. *R*^2^	0.107	0.271	0.352

Note: The standard error in parenthesis. ***,**, and * indicate statistically significant at 1%, 5%, and 10%, respectively.

As shown in columns (1)–(3), the regression coefficient of digital trade is significantly positive in the three industries of labor, capital, and technology-intensive, with the largest in the technology-intensive industry. This indicates that the development of digital trade has the most obvious effect on the upgrading of the export technology complexity of the technology-intensive manufacturing industry. There are a number of possible reasons for this. First, the current development of digital trade is characterized by all-sided and all-directional penetration, which leads to significant increases in the export technology complexity of manufacturing industries. Second, the technology-intensive manufacturing industry is characterized by electronic equipment, special equipment, etc. The digital technology application threshold and technical versatility of digital trade play synergistic roles, promoting export technology complexity.

### Mediating effects

To further clarify the internal mechanism of digital trade affecting the export technology complexity of the manufacturing industry, we verified how this occurs through intermediary effects. The regression results are shown in Table 9.

**Table 9:** Regression results of mediating effects of digital trade on the export technology complexity of the manufacturing industry.

	(1)	(2)	(3)	(4)	(5)	(6)
	ln*etc*	ln*pat*	ln*etc*	ln*etc*	*hum*	ln*etc*
ln*pat*			0.277***			
			(0.049)			
*hum*						0.138**
						(0.057)
*dt*	1.410***	0.722*	1.209***	1.410***	0.880***	1.181***
	(0.320)	(0.381)	(0.305)	(0.320)	(0.193)	(0.329)
ln*ci*	-0.248***	-0.189**	-0.196***	-0.248***	-0.116***	-0.218***
	(0.067)	(0.079)	(0.064)	(0.067)	(0.040)	(0.067)
ln*lp*	0.313***	0.356***	0.214**	0.313***	0.267***	0.243**
	(0.113)	(0.135)	(0.108)	(0.113)	(0.068)	(0.115)
*open*	0.037***	0.043***	0.025***	0.037***	0.017***	0.033***
	(0.009)	(0.010)	(0.008)	(0.009)	(0.005)	(0.009)
*mar*	0.199***	0.551***	0.046	0.199***	0.141***	0.163***
	(0.034)	(0.040)	(0.042)	(0.034)	(0.020)	(0.036)
*_cons*	7.383***	3.883**	6.307***	7.383***	6.030***	5.820***
	(1.304)	(1.552)	(1.248)	(1.304)	(0.784)	(1.428)
*N*	300	300	300	300	300	300
Hausman	43.00***	23.40***	44.54***	43.00***	10.54*	47.26***
adj. *R*^2^	0.387	0.623	0.451	0.387	0.454	0.399

Note: The standard error in parenthesis. ***,**, and * indicate statistically significant at 1%, 5%, and 10%, respectively.

#### (1) Technological innovation effect

As shown in Table 9, column (1) illustrates the direct impact of digital trade on the export technology complexity of the manufacturing industry. It can be seen that the impact coefficient of digital trade is significantly positive, with a coefficient of 1.410. Column (2) illustrates the impact of digital trade on technological innovation, with a significant positive coefficient of 0.722. Column (3) illustrates the impact of digital trade and technological innovation on the export technology complexity of the manufacturing industry. As shown, the intermediary variable of technological innovation has a significant positive impact on the export technology complexity of the manufacturing industry, with a coefficient of 0.277. Digital trade has a significant positive impact on the export technology complexity of the manufacturing industry, with a coefficient of 1.209, indicating that the intermediary variable plays a part in the intermediary effect. It shows that the core explanatory variable, digital trade development level, partially affects the export technology complexity of the manufacturing industry through technological innovation. The intermediary effect value is 0.1419, which means that 14.19% of the promotion of digital trade on the export technology complexity of the manufacturing industry is realized through technological innovation. Therefore, Hypothesis 2a is supported.

#### (2) Human capital effect

As shown in Table 9, column (4) illustrates the direct impact of digital trade on the export technology complexity of the manufacturing industry. It can be seen that the impact coefficient of digital trade is significantly positive, with a coefficient of 1.410. Column (5) examines the impact of digital trade on human capital, with a significant positive coefficient of 0.880. Column (6) examines the impact of digital trade and human capital on the export technology complexity of the manufacturing industry. The intermediary variable of human capital has a significant positive impact on the export technology complexity of the manufacturing industry, with a coefficient of 0.138. Digital trade has a significant positive impact on the export technology complexity of the manufacturing industry, with a coefficient of 1.181, indicating that the intermediary variable plays a part in the intermediary effect. It shows that the core explanatory variable, digital trade development level, partially affects the export technology complexity of the manufacturing industry through human capital. The intermediary effect value is 0.0861, which means that 8.61% in the promotion of digital trade on the export technology complexity of the manufacturing industry is realized through the human capital effect. Therefore, Hypothesis 2b is supported.

The impact coefficient of industrial capital intensity is significantly negative, which may be due to the phased development of the export technology complexity of the manufacturing industry. In the early stages, the manufacturing industry is mostly driven by capital forces. Manufacturing industry exports have been more prone to being innovation-driven since 2011, and the power of transforming capital into export technology complexity has gradually weakened. The impact coefficients of openness to foreign trade and to the domestic market are both positive, which may be because an increase in foreign direct investment can produce learning and imitation effects, and improve the quality of export products. The higher the degree of openness to the domestic market, the more effective the allocation and efficient circulation of resources. This is conducive to improving the efficiency of product production and circulation, and ultimately enhances the technology complexity of the manufacturing industry.

### Robustness of the mediation test

The robustness of the mesomeric effect is tested primarily by adding control variables and replacing explained variables. A new industrial wage level (wl) control variable was added for robustness testing, and the results are shown in Table 10. By replacing the core explanatory variables and using the entropy-weighted TOPSIS method mentioned earlier, the core explanatory variables are calculated. The regression results are shown in Table 11. The results of the robustness test are basically consistent with the results of the intermediary test, and the model has a relatively high degree of fit. The core explanatory variable, the level of digital trade development, is consistent with the significance and coefficient of the impact of technological innovation and human capital on manufacturing technology complexity through the mediating variable of technological innovation and human capital, indicating that the research results are relatively reliable.

**Table 10:** Results of the robustness of the mediation test: Adding control variables.

	(1)	(2)	(3)	(4)	(5)	(6)
	ln*etc*	ln*pat*	ln*etc*	ln*etc*	*hum*	ln*etc*
ln*pat*			0.277***			
			(0.049)			
*hum*						0.259**
						(0.101)
*dt*	1.390***	0.658*	1.208***	1.390***	0.879***	1.162***
	(0.325)	(0.386)	(0.309)	(0.325)	(0.195)	(0.334)
ln*ci*	-0.258***	-0.223***	-0.196***	-0.258***	-0.116***	-0.228***
	(0.072)	(0.085)	(0.069)	(0.072)	(0.043)	(0.072)
ln*lp*	0.290**	0.281*	0.212*	0.290**	0.266***	0.221*
	(0.128)	(0.152)	(0.122)	(0.128)	(0.077)	(0.129)
*open*	0.037***	0.042***	0.025***	0.037***	0.017***	0.032***
	(0.009)	(0.010)	(0.008)	(0.009)	(0.005)	(0.009)
*mar*	0.199***	0.549***	0.047	0.199***	0.141***	0.162***
	(0.034)	(0.040)	(0.042)	(0.034)	(0.020)	(0.036)
ln*wl*	0.043	0.143	0.003	0.043	0.001	0.043
	(0.112)	(0.132)	(0.106)	(0.112)	(0.067)	(0.110)
*_cons*	7.356***	3.792**	6.305***	7.356***	6.029***	5.793***
	(1.308)	(1.554)	(1.252)	(1.308)	(0.786)	(1.432)
*N*	300	300	300	300	300	300
Hausman	39.24***	40.94***	46.14***	39.24***	17.18***	44.13***
adj. *R*^2^	0.385	0.624	0.449	0.385	0.452	0.397

Note: The standard error in parenthesis. ***,**, and * indicate statistically significant at 1%, 5%, and 10%, respectively.

**Table 11:** Results of the robustness of mediation test: Replacing explanatory variables.

	(1)	(2)	(3)	(4)	(5)	(6)
	ln*etc*	ln*pat*	ln*etc*	ln*etc*	*hum*	ln*etc*
ln*pat*			0.280***			
			(0.049)			
*hum*						0.259**
						(0.102)
*dt*	1.198***	0.591*	1.033***	1.198***	0.846***	0.979***
	(0.287)	(0.341)	(0.273)	(0.287)	(0.171)	(0.297)
ln*ci*	-0.268***	-0.199**	-0.212***	-0.268***	-0.129***	-0.234***
	(0.067)	(0.079)	(0.064)	(0.067)	(0.040)	(0.067)
ln*lp*	0.343***	0.372***	0.239**	0.343***	0.283***	0.270**
	(0.113)	(0.134)	(0.108)	(0.113)	(0.067)	(0.116)
*open*	0.037***	0.043***	0.025***	0.037***	0.017***	0.032***
	(0.009)	(0.010)	(0.009)	(0.009)	(0.005)	(0.009)
*mar*	0.213***	0.560***	0.056	0.213***	0.142***	0.176***
	(0.032)	(0.038)	(0.041)	(0.032)	(0.019)	(0.035)
*_cons*	7.184***	3.761**	6.131***	7.184***	5.991***	5.631***
	(1.302)	(1.546)	(1.244)	(1.302)	(0.775)	(1.427)
*N*	300	300	300	300	300	300
Hausman	44.48***	25.32***	45.49***	44.48***	10.30*	48.58***
adj. *R*^2^	0.382	0.623	0.449	0.382	0.461	0.395

Note: The standard error in parenthesis. ***,**, and * indicate statistically significant at 1%, 5%, and 10%, respectively.

### Further analysis

Based on Hansen’s theory, industrial structure upgrading (ind) is taken as the threshold variable to carry out the effect test in turn, and the bootstrap method is adopted to repeatedly sample 500 times to obtain the asymptotic distribution of the F-value, corresponding P-value, and critical value (Table 12). The single threshold value of the threshold variable is 1.4591, and is significant at the 5% level. Both the double threshold and the triple threshold failed to pass the significance test. This shows that the assumption of rejecting the linear relationship with industrial structure upgrading as the threshold variable has a single threshold effect, and the effect of digital trade on the export technology complexity of the manufacturing industry through industrial structure upgrading has a nonlinear relationship, which is not static. Therefore, Hypothesis 3 is supported.

**Table 12:** Significance test, estimated value, and confidence interval of the threshold.

Number of threshold	Threshold value	F value	P value	BS times	Critical value			95% confidence interval
					10%	5%	1%	
Single	1.4591	27.23	0.048	500	24.4348	31.9391	40.0785	[1.4445, 1.4594]
Double	0.9201	8.64	0.564	500	19.1994	22.8139	30.8608	[0.8679, 0.9207]
Triple	1.5293	7.23	0.676	500	21.7427	25.0473	42.2682	[1.5248, 1.5328]

**Fig 2:**
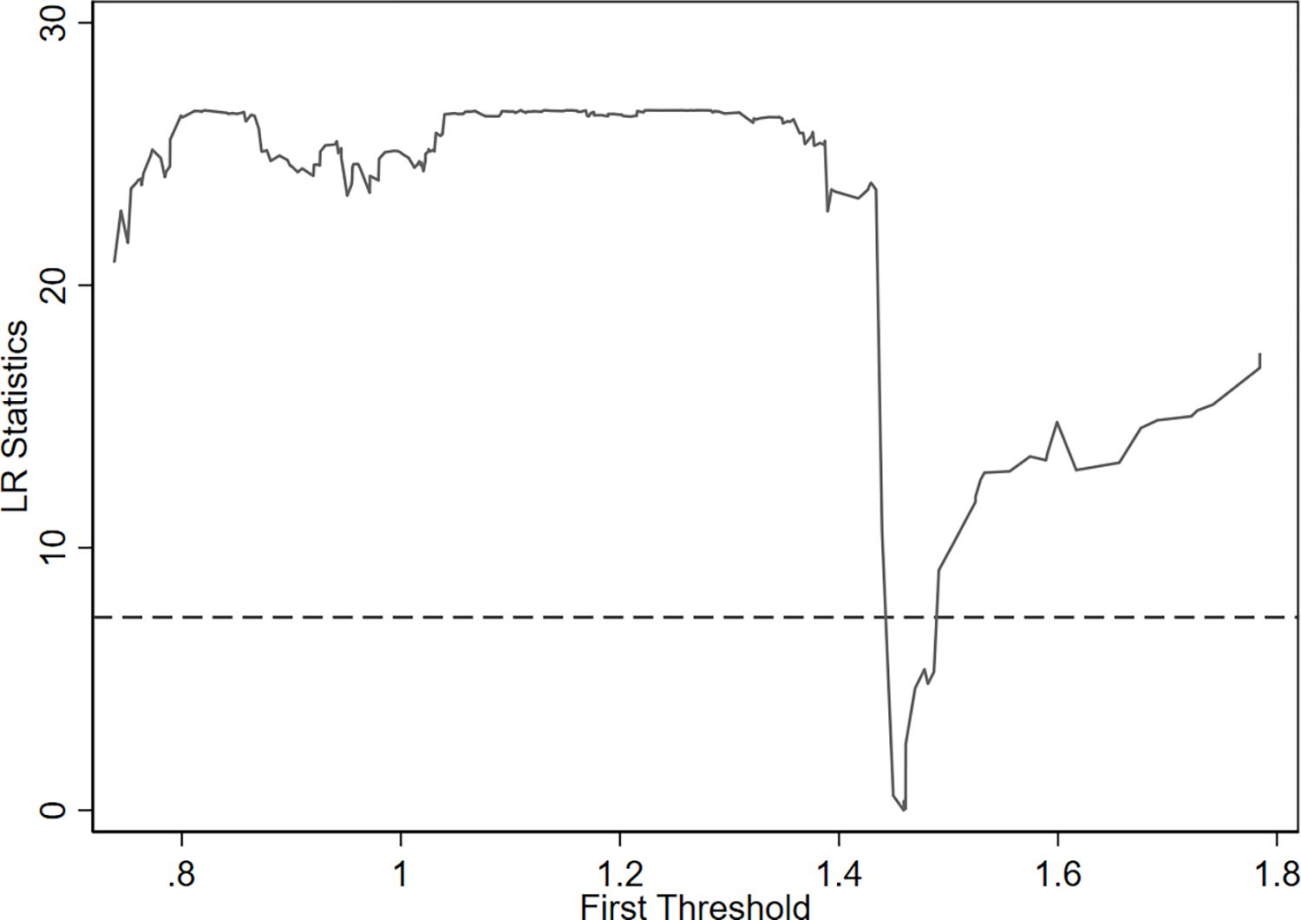
LR diagram with *ind* as the threshold variable.

To better understand the threshold estimation and test the authenticity of the confidence interval, we drew a likelihood ratio function diagram (Fig 2). The corresponding threshold estimates (ind) are 1.4445 and 1.4594, respectively, when the LR value of the likelihood ratio statistics is equal to 0. Some threshold variables below the critical value of the 5% significance level (shown by the dotted line) pass the validity test.

According to the threshold effect regression results (Table 13), the fitting degree is 0.482, and the fitting effect is good. When the industrial structure upgrading is less than or equal to 1.4591, the impact coefficient is 0.788. It is significant at the 1% level, indicating that digital trade plays a certain role in promoting the export technology complexity of the manufacturing industry. When the industrial structure upgrading is greater than 1.4591, the impact coefficient is 2.852, and it is significant at the 1% level. The impact of digital trade on the export technology complexity of the manufacturing industry is still positive at this point, and the promotion role is continually strengthened, indicating that this impact is significantly positive. When industrial structure upgrading breaks through a certain threshold, the promotion becomes stronger.

**Table 13:** Threshold regression results.

	ln*etc*
*ind*≤1.4591	0.788***
	(0.286)
*ind*>1.4591	2.852***
	(0.042)
Control variable	YES
*_cons*	6.808***
	(1.347)
*N*	300
F value	37.25***
adj. *R*^2^	0.482

Note: The standard error in parenthesis. ***,**, and * indicate statistically significant at 1%, 5%, and 10%, respectively.

The situation in which the industrial structure upgrading of each province has reached the threshold value over the years is shown in Table 14. It can be seen that the number of provinces and cities that have reached the threshold value has increased over time, especially since 2016. In general, the provinces and cities that have reached the threshold value are mainly concentrated in more developed eastern coastal areas, such as Beijing, Shanghai, Jiangsu, and Guangdong. However, some provinces and cities in the central and western regions, such as Gansu, Anhui, Sichuan, and Guizhou, have also reached the threshold value in recent years.

**Table 14:** Provinces that have reached the threshold value of industrial structure upgrading in 2011–2020.

2011	2012	2013	2014	2015	2016	2017	2019	2020
Beijing	Beijing	Beijing	Beijing	Beijing	Beijing	Beijing	Beijing	Beijing
Jiangsu	Shanghai	Shanghai	Shanghai	Shanghai	Tianjin	Tianjin	Tianjin	Tianjin
	Jiangsu	Jiangsu	Jiangsu	Jiangsu	Guangdong	Guangdong	Guangdong	Guangdong
					Shanghai	Shanghai	Shanghai	Shanghai
					Jiangsu	Jiangsu	Jiangsu	Jiangsu
					Zhejiang	Zhejiang	Zhejiang	Zhejiang
					Gansu	Gansu	Gansu	Gansu
						Anhui	Anhui	Anhui
							Sichuan	Sichuan
							Liaoning	Liaoning
								Guizhou

## Conclusions and implications

### Conclusions

The digital trade development level and the export technology complexity of the manufacturing industry were measured in 30 provinces and cities of China from 2011 to 2020, and then the theoretical path hypothesis that digital trade affects the export technology complexity of the manufacturing industry was verified using models such as benchmark regression, mediating effect regression, and panel threshold. The results are as follows. The direct impact of digital trade on the export technology complexity of the manufacturing industry in China plays a promotional role, with significant regional heterogeneity, and with the most obvious promotion in technology-intensive manufacturing. Technological innovation and human capital play a reallocation role in the process of digital trade, affecting the technological complexity of manufacturing exports, with mediating effects of 14.19% and 8.61%, respectively. Digital trade development affects the export technology complexity of the manufacturing industry through industrial structure upgrading, and there is a nonlinear relationship. The effect of digital trade on the export technology complexity of the manufacturing industry through industrial structure upgrading has a nonlinear relationship, which is not static. The single threshold value of industrial structure upgrading is 1.4591. The provinces and cities that reached the threshold in 2011–2020 are mainly concentrated in the developed eastern coastal areas, but some provinces and cities in the central and western regions have also reached the threshold in recent years.

### Implications

First, investment in digital infrastructure construction should continue to increase, with digital trade used to promote the complexity of manufacturing exports. Urban network upgrading and rural broadband coverage provide solid support for the development of digital trade. In this regard, the government should increase the construction of digital infrastructure to reduce the cost of digital trade, improve the convenience of trade, and ultimately improve the technical complexity of manufacturing exports.

Second, the differentiated development of digital trade in the eastern, central, and western regions, focusing on the export of technology-intensive manufacturing, emphasizes the intermediary role of technological innovation and human capital. In view of the reality of the development of digital trade in different regions of China, policy makers should encourage and support digital trade in the central and western regions. For example, investment could be increased in the construction of digital infrastructure in backward areas, and high-quality production factors, such as advanced technology and digital technology talents, could be supported and attracted in these areas. It is necessary to improve the technical content of manufacturing exports and reduce the trade dilemma of “two ends outside.” It is also necessary to increase research and development investment in the field of digital trade technology and reconfigure the elements effectively, such as creating a safe digital trade environment and avoiding problems such as “violating user privacy and threatening the security of corporate and personal data.” It is also necessary to train a group of talents in digital trade, manufacturing export, and other fields in accordance with professional standards, and give full play to the cumulative effect of human capital.

Third, promoting the upgrading of the industrial structure scientifically and effectively helps improve the complexity of export technology. Government departments need to strengthen the overall layout and planning of the industry and promote the upgrading of the industrial structure. For example, they could build a number of benchmark demonstration enterprises in the field of digital trade, give full play to the leading role of enterprises in digital technology research and development and production and operation, and steadily promote and coordinate the upgrading of industrial structure so as to build a bridge between digital trade and the technical complexity of manufacturing exports.

The present study has some limitations that future studies could address. First, when measuring the technical complexity of manufacturing exports, Hausmann’s method cannot distinguish trade patterns, so it can only be measured using the general form of export trade. Second, while exploring the indirect mechanism by which digital trade affects the technical complexity of manufacturing exports, other mechanisms could also be explored in depth. Finally, future research could explore the relationship between digital trade and the technical complexity of manufacturing exports at the firm level, which is currently difficult to achieve due to limitations in data access.

## Supporting information

S1 File (XLS)

## References

[1] Abeliansky AL, Hilbert M. Digital technology and international trade: Is it the quantity of subscriptions or the quality of data speed that matters? Telecommunications Policy. 2017; 41(1): 35–48. 10.1016/j.telpol.2016.11.001

[2] Kumar V, Ramachandran D, Kumar B. The influence of new-age technologies: A research agenda. Journal of Business Research. 2019; 125: 864–877. 10.1016/j.jbusres.2020.01.007

[3] Carlsson B. The digital economy: What is new and what is not? Structural Change and Economic Dynamics. 2004; 15(3): 245–264. 10.1016/j.strueco.2004.02.001

[4] Elsig M, Klotz S. Initiator conditions and the diffusion of digital trade-related provisions in PTAs. International Interactions. 2022; 48(2): 292–308. 10.1080/03050629.2022.2004137

[5] Weber RH. Digital trade in WTO-Law-Taking stock and looking ahead. Asian Journal of WTO & International Health Law and Policy. 2010; 5(1): 1–24. 10.2139/ssrn.1578139

[6] Deardorff AV. Sensitive sectors in free trade agreements. East Asian Economic Review. 2018; 22(4): 403–425. 10.11644/KIEP.EAER.2018.22.4.349

[7] USITC. Digital trade in the U.S. and global economies, Part 1. 2013; https://usitc.gov/publications/332/pub4415.pdf

[8] MGI. Digital globalization: the new era of global flows. 2016; 03. https://www.mckinsey.com/capabilities/mckinsey-digital/our-insights/digital-globalization-the-new-era-of-global-flows

[9] Ma S, Guo J, Zhang H. Policy analysis and development evaluation of digital trade: An International Comparison. China & World Economy. 2019; 27(3): 49–75. 10.1111/cwe.12280

[10] Hausmann R, Hwang J, Rodrik D. What you export matters. Journal of Economic Growth. 2007; 12: 1–25. 10.1007/s10887-006-9009-4

[11] Fan Z, Anwar S, Huang S. Cultural diversity and export sophistication. International Review of Economics & Finance. 2018; 58(C): 508–522. 10.1016/j.iref.2018.05.008

[12] Thorbecke W, Salike N. Export sophistication and trade elasticities. Journal of Asian Economic Integration. 2020; 2(1): 7–26. 10.1177/2631684620910276

[13] Su X, Anwar S, Zhou Y, Tang X. Services trade restrictiveness and manufacturing export sophistication. The North American Journal of Economics and Finance. 2020; 51(C): 101058. 10.1016/j.najef.2019.101058

[14] Lv C, Song J, Lee CC. Can digital finance narrow the regional disparities in the quality of economic growth? Evidence from China. Economic Analysis and Policy. 2022; 76: 502–521. 10.1016/j.eap.2022.08.022

[15] Lee CC, Lou R, Wang F. Digital financial inclusion and poverty alleviation: Evidence from the sustainable development of China. Economic Analysis and Policy. 2023; 77: 418–434. 10.1016/j.eap.2022.12.004

[16] Aguerre C. Digital trade in Latin America: Mapping issues and approaches. Digital Policy Regulation and Governance. 2019; 21(1): 2–18. 10.1108/DPRG-11-2018-0063

[17] Bas M. Does services liberalization affect manufacturing firms’ export performance? Evidence from India. Journal of Comparative Economics. 2014; 42(3): 569–589. 10.1016/j.jce.2013.06.005

[18] Hiranya KN, Liu L. Information and communications technology (ICT) and services trade. Information Economics and Policy. 2017; (41): 81–87. 10.1016/j.infoecopol.2017.06.003

[19] Zhou R, Tang D, Da D, Chen W, Kong L, Boamah V. Research on China’ s manufacturing industry moving towards the middle and high-end of the GVC driven by digital economy. Sustainability. 2022; 14(13): 1–30. 10.3390/su14137717

[20] Jiang M, Jia P. Does the level of digitalized service drive the global export of digital service trade? Evidence from global perspective. Telematics and Informatics. 2022; (72): 101853. 10.1016/j.tele.2022.101853

[21] Obashi A, Kimura F. New developments in international production networks: Impact of digital technologies. Asian Economic Journal. 2021; 35(2): 115–141. 10.1111/asej.12240

[22] Mccaig B, Pavcnik N. Export Markets and Labor Allocation in a Low-Income Country. American Economic Review. 2018; 108(7): 1899–1941. 10.1257/aer.20141096

[23] Jongwanith J, Kohpaiboon A, Obashi A. Technological advancement, import penetration and labour markets: Evidence from Thailand. World Development. 2022; (151): 105746. 10.1016/j.worlddev.2021.105746

[24] Xing Z. The impacts of information and communications technology (ICT) and E-commerce on bilateral trade flows. International Economics and Economic Policy. 2018; 15(3): 565–586. 10.1007/s10368-017-0375-5

[25] Goldfarb A, Tucker C. Digital economics. Journal of Economic Literature. 2019; 57: 3–43. http://www.nber.org/papers/w23684

[26] Hottman CJ, Redding RW, Weinstein DE. Quantifying the sources of firm heterogeneity. The Quarterly Journal of Economics. 2016; 131(3): 1291–1364. 10.1093/qje/qjw012

[27] Lin C, Xiao S, Yin Z. How do industrial robots applications affect the quality upgrade of Chinese export trade? Telecommunications Policy. 2022; 46(10): 102425. 10.1016/j.telpol.2022.102425

[28] Rodríguez-Crespo E, Martínez-Zarzoso I. The effect of ICT on trade: Does product complexity matter? Telematics and Informatics. 2019; 41(C): 182–196. 10.1016/j.tele.2019.05.001

[29] Meltzer JP. Governing digital trade. World Trade Review. 2019; 18(1): 23–48. 10.1017/S1474745618000502

[30] Jiang X, Luo L. Globalization of services in the internet age: A new engine, acceleration and major power competitiveness. Social Sciences in China. 2020; 41(4): 5–23. 10.1080/02529203.2020.1844430

[31] Elliott RJR, Jabbour L, Vanino E. Innovation and the creative destruction of trade: A study of the intensive and extensive margins of trade for French firms. Oxford Bulletin of Economics and Statistics. 2020; 82(1): 180–208. 10.1111/obes.12324

[32] Gkypali A, Love JH, Roper S. Export status and SME productivity: Learning-to-export versus learning-by-exporting. Journal of Business Research. 2021; 128(C): 486–498. 10.1016/j.jbusres.2021.02.026

[33] Bernard AB, Jensen JB, Redding SJ, Schott PK. Firms in international trade. Journal of Economic Perspectives. 2007; 21(3): 105–130. 10.1257/jep.21.3.105

[34] Mikalef P, Pateli A. Information technology-enabled dynamic capabilities and their indirect effect on competitive performance: Findings from PLS-SEM and fsQCA. Journal of Business Research. 2017; 70: 1–16. 10.1016/j.jbusres.2016.09.004

[35] Ma S, Chai Y, Zhang H. Rise of cross-border E-commerce exports in China. China & World Economy. 2018; 26(3): 63–87. 10.1111/cwe.12243

[36] Visser R. The effect of the internet on the margins of trade. Information Economics and Policy. 2019; 46: 41–54. 10.1016/j.infoecopol.2018.12.001

[37] Lee CC, He ZW, Wen H. The impact of digitalization on green economic efficiency: Empirical evidence from city-level panel data in China. Energy & Environment; 2022; 0(0). 10.1177/0958305X221124225

[38] You S, Zhou KZ, Jia L. How does human capital foster product innovation? The contingent roles of industry cluster features. Journal of Business Research. 2021; 130(C): 335–347. 10.1016/j.jbusres.2021.03.046

[39] Costinot A. On the origins of comparative advantage. Journal of International Economics. 2009; 77(2): 255–264. 10.1016/j.jinteco.2009.01.007

[40] Hu Y, Zhou HQ, Yan B, Zou Z, Li Y. An assessment of China’s digital trade development and influencing factors. Frontiers in Psychology. 2022; 13: 837885. doi: 10.3389/fpsyg.2022.837885 35558693 PMC9087175

[41] Metcalfe B. Metcalfe’s law after 40 years of ethernet. Computer. 2013; 46(12): 26–31. 10.1109/MC.2013.374

[42] Hong A, Cheng C. The study on affecting factors of regional marine industrial structure upgrading. International Journal of System Assurance Engineering and Management. 2016; 7: 213–219. 10.1007/s13198-016-0440-4

[43] Fatum R, Liu R, Tong J, Xu J. Beggar thy neighbor or beggar thy domestic firms? Evidence from 2000 to 2011 Chinese customs data. Journal of International Economics. 2018; (115): 16–29. 10.1016/j.jinteco.2018.07.007

[44] Bas M, Strauss-Kahn V. Input-trade liberalization, export prices and quality upgrading. Journal of International Economics. 2015; 95(2): 250–262. 10.1016/j.jinteco.2014.12.005

[45] Collard-Wexler A, Loecker JD. Reallocation and technology: Evidence from the US steel industry. American Economic Review. 2015; 105(1): 131–71. 10.1257/aer.2013009029543407

[46] Helpman E, Melitz MJ, Yeaple SR. Export versus FDI with heterogeneous firms. American Economic Review. 2004; 94(1): 300–316. 10.1257/000282804322970814

[47] Tombe T, Zhu X. Trade, migration, and productivity: A quantitative analysis of China. American Economic Review. 2019; 109(5): 1843–1872. https://doi.org/doi:10.1257/aer.20150811

[48] Nguyen DX. Trade liberalization and export sophistication in Vietnam. The Journal of International Trade & Economic Development. 2016; 25(8): 1071–1089. 10.1080/09638199.2016.1179778

[49] Hansen BE. Threshold effects in non-dynamic panels: Estimation, testing, and inference. Journal of Econometrics. 1999; 93(2): 345–368. 10.1016/S0304-4076(99)00025-1

